# The genome sequence of the Oak Polypore, *Buglossoporus quercinus* (Schrad.) Kotl. & Pouzar

**DOI:** 10.12688/wellcomeopenres.24714.1

**Published:** 2025-08-22

**Authors:** Richard Wright, Kieran Woof, Ester Gaya, Royal Botanic Gardens Kew Genome Acquisition Lab, Wellcome Sanger Institute Tree of Life Management, Samples and Laboratory team, Wellcome Sanger Institute Scientific Operations: Sequencing Operations, Wellcome Sanger Institute Tree of Life Core Informatics team, Tree of Life Core Informatics collective, Darwin Tree of Life Consortium

**Affiliations:** 1 Cardiff University, Cardiff, Wales, UK; 2 Royal Botanic Gardens Kew, Richmond, England, UK

**Keywords:** Buglossoporus quercinus, Oak Polypore, genome sequence, chromosomal, Polyporales

## Abstract

We present a genome assembly from a specimen of *Buglossoporus quercinus* (Oak Polypore; Basidiomycota; Agaricomycetes; Polyporales; Fomitopsidaceae). The genome sequence has a total length of 37.43 megabases. Most of the assembly (99.81%) is scaffolded into 13 chromosomal pseudomolecules. The mitochondrial genome has also been assembled, with a length of 70.88 kilobases.

## Species taxonomy

Eukaryota; Opisthokonta; Fungi; Dikarya; Basidiomycota; Agaricomycotina; Agaricomycetes; Agaricomycetes incertae sedis; Polyporales; Fomitopsidaceae; *Buglossoporus*; *Buglossoporus quercinus* (Schrad.) Kotl. & Pouzar (NCBI:txid1522478)

## Background

**Figure 1.**
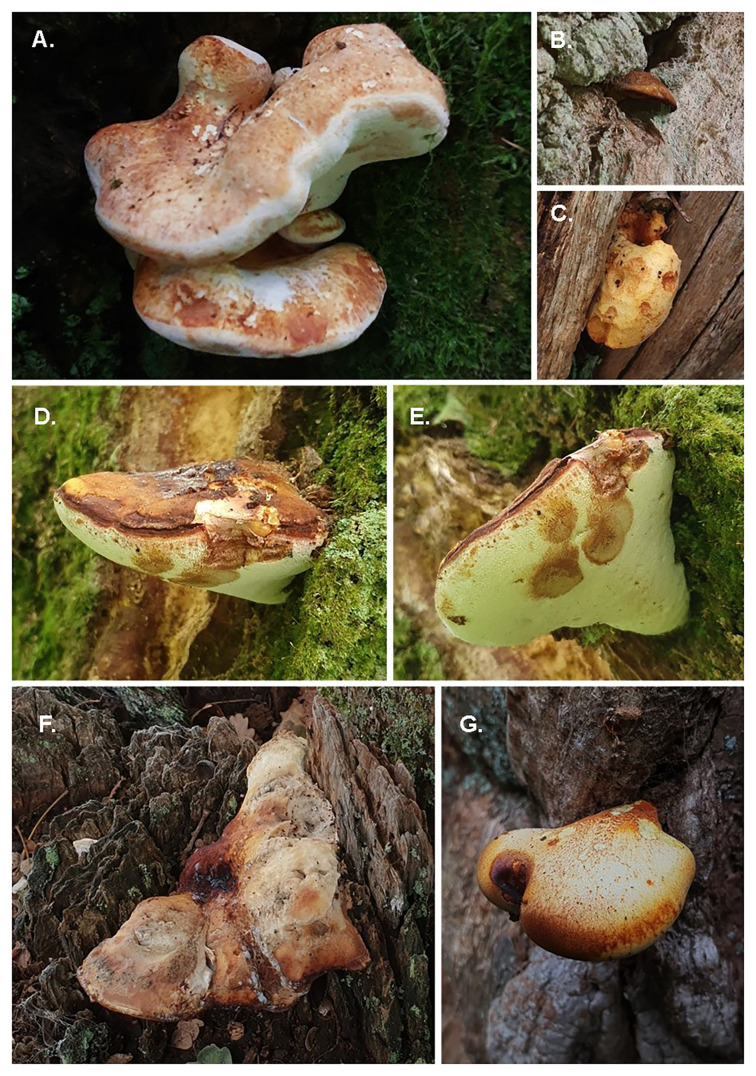
Sporocarps of *B. quercinus*, **A.** mature sporocarp, **B.** Old sporocarp, **C.** pileus chewed away by invertebrates, **D & E.** mature sporocarp showing leathery pileus and brown bruising of pores, **F.** old sprocarp, **G.** young sporocarp showing fibrils on pileus.

*Buglossoporus quercinus* (oak polypore) is a poroid fungus which produces fleshy, pale white to cream, hoof- to tongue-shaped, annual bracket sporocarps ( Figure 1), with a yellow, ageing to brown pileus surface which is initially velutinous, becoming smooth and glabrous with age ( Læssøe & Petersen, 2019). The outer flesh and context become vinaceous when bruised, eventually turning brown. The pore layer is white, entirely smooth and contains 2 to 4 round pores per mm ( Ryvarden & Melo, 2017). The sporocarp is rubbery and tough throughout, often resembling a more yellowish *Fomitopsis betulina*, and can be mistaken for *Laetiporus sulphureus* in its very early or late stages. The hyphal system is dimitic in the context and monomitic in the trama, with hyaline to pale brown, clamped, large, branching generative hyphae throughout. The vegetative hyphae are thick-walled and non-septate ( Ryvarden & Melo, 2017). Spores are thin-walled, hyaline, cylindrical, fusiform, inamyloid, measuring 6–8 × 2.5–3.5 μm, and they produce a white spore deposit.

This rare brown-rot fungus, regarded as a flagship for fungus conservation ( Farjon, 2017), is obligate on oak ( Mitchell *et al.*, 2019; Wright, 2024), and is most commonly only found on older veteran oaks or on sites with longstanding continuity of oak habitat. Trees that support fruiting are also generally found in open situations, often exposed to extremes of temperature and dehydration, which indicates its adaption to these intense conditions ( Roberts, 2002). Sporocarps are produced ahead of the main sporulating season, from June to September and, due to their relatively tough nature, can persist up to a year in extreme cases.

It is protected under Schedule 8 of the Wildlife and Countryside Act 1981, on the list of rare and most threatened species under section 41 and 42 of the Natural Environment and Rural Communities Act 2006, and is globally Red Listed as vulnerable ( IUCN, 2021).

This species is found in temperate Europe and Asia, although it is considered rare throughout this range ( Crockatt *et al.*, 2010; Roberts, 2002). The UK has the highest number of known sites, showing a distinctly south-eastern distribution with very few Scottish, Cornish, or Welsh records. It is currently not thought to be present in Ireland (unpublished data from historical Fungal Records Database of Britain and Ireland; Cannon, 1998; The British Mycological Society, 2009; and recent record data as part of Lost and Found Fungi project at the Royal Botanic Gardens, Kew).

*B. quercinus* is a key species of conservation concern, which is threatened by geographical isolation causing inbreeding ( Crockatt *et al.*, 2010). Interventions such as translocation are hopeful methods for the protection of *B. quercinus* ( Wright, 2024), and the genome produced here has the potential to help us better understand the mating genetics of this species to inform future conservation actions.

We present a chromosome-level genome sequence for *Buglossoporus quercinus*, based on a specimen from Cardiff University, Cardiff, Wales, UK. The specimen was sequenced as part of the Darwin Tree of Life project.

## Genome sequence report

### Sequencing data

**Figure 2.**
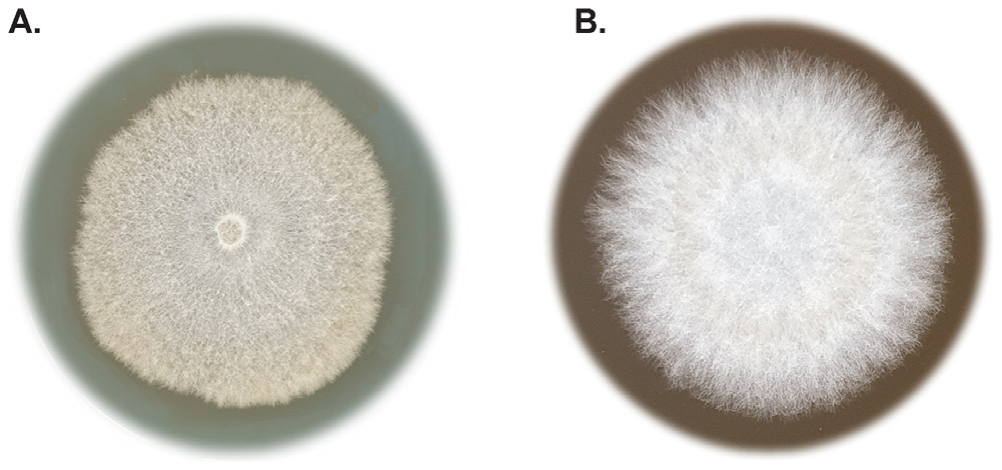
*Buglossoporus quercinus* cultures **A**. 40 days growth on malt extract agar. **B**. A close-up of 20 days growth on malt extract agar with 1% lactic acid.

The genome of a specimen of *Buglossoporus quercinus* ( Figure 1 and Figure 2) was sequenced using Pacific Biosciences single-molecule HiFi long reads, generating 24.31 Gb (gigabases) from 2.46 million reads. GenomeScope analysis of the PacBio HiFi data estimated the haploid genome size at 35.44 Mb, with a heterozygosity of 0.45% and repeat content of 21.08%. These values provide an initial assessment of genome complexity and the challenges anticipated during assembly. Based on this estimated genome size, the sequencing data provided approximately 612.0x coverage of the genome. Chromosome conformation Hi-C sequencing produced 133.14 Gb from 881.71 million reads. Table 1 summarises the specimen and sequencing information.

**Table 1.** Specimen and sequencing data for *Buglossoporus quercinus*.

Project information			
**Study title**	Buglossoporus quercinus (oak polypore)		
**Umbrella BioProject**	PRJEB65737		
**Species**	*Buglossoporus quercinus*		
**BioSpecimen**	SAMEA13743897		
**NCBI taxonomy ID**	1522478		
Specimen information			
**Technology**	**ToLID**	**BioSample** **accession**	**Organism part**
**PacBio long read sequencing**	gfBolQuer1	SAMEA13744693	mycelium
**Hi-C sequencing**	gfBolQuer1	SAMEA13744693	mycelium
**RNA sequencing**	gfBolQuer1	SAMEA13744692	mycelium
Sequencing information			
**Platform**	**Run accession**	**Read count**	**Base count (Gb)**
**Hi-C Illumina NovaSeq 6000**	ERR12035318	8.82e+08	133.14
**PacBio Sequel IIe**	ERR12015777	2.46e+06	24.31
**RNA Illumina NovaSeq 6000**	ERR12245597	7.96e+07	12.02

### Assembly statistics

The primary haplotype was assembled, and contigs corresponding to an alternate haplotype were also deposited in INSDC databases. The assembly was improved by manual curation, which corrected 20 misjoins or missing joins and removed three haplotypic duplications. These interventions reduced the total assembly length by 0.71%, the scaffold count by 30.0% and also decreased the scaffold N50 by 15.4%. The final assembly has a total length of 37.43 Mb in 13 scaffolds, with 81 gaps, and a scaffold N50 of 2.93 Mb ( Table 2).

**Table 2.** Genome assembly data for *Buglossoporus quercinus*.

Genome assembly		
Assembly name	gfBolQuer1.1	
Assembly accession	GCA_964035675.1	
*Alternate haplotype* *accession*	*GCA_964035665.1*	
Assembly level for primary assembly	chromosome	
Span (Mb)	37.43	
Number of contigs	94	
Number of scaffolds	13	
Longest scaffold (Mb)	5.34	
**Assembly metric**	**Measure**	*Benchmark*
Contig N50 length	0.83 Mb	*≥ 1 Mb*
Scaffold N50 length	2.93 Mb	*= chromosome N50*
Consensus quality (QV)	Primary: 64.7; alternate: 66.9; combined: 65.6	*≥ 40*
*k*-mer completeness	Primary: 88.76%; alternate: 86.47%; combined:98.85 %	*≥ 95%*
BUSCO *	C:95.1%[S:94.1%,D:1.0%], F:0.8%,M:4.2%,n:4,464	*S > 90%; D < 5%*
Percentage of assembly mapped to chromosomes	99.81%	*≥ 90%*
Organelles	Mitochondrial genome: 70.88 kb	*complete single alleles*

* BUSCO scores based on the polyporales_odb10 BUSCO set using version 5.5.0. C = complete [S = single copy, D = duplicated], F = fragmented, M = missing, n = number of orthologues in comparison.

**Figure 3.**
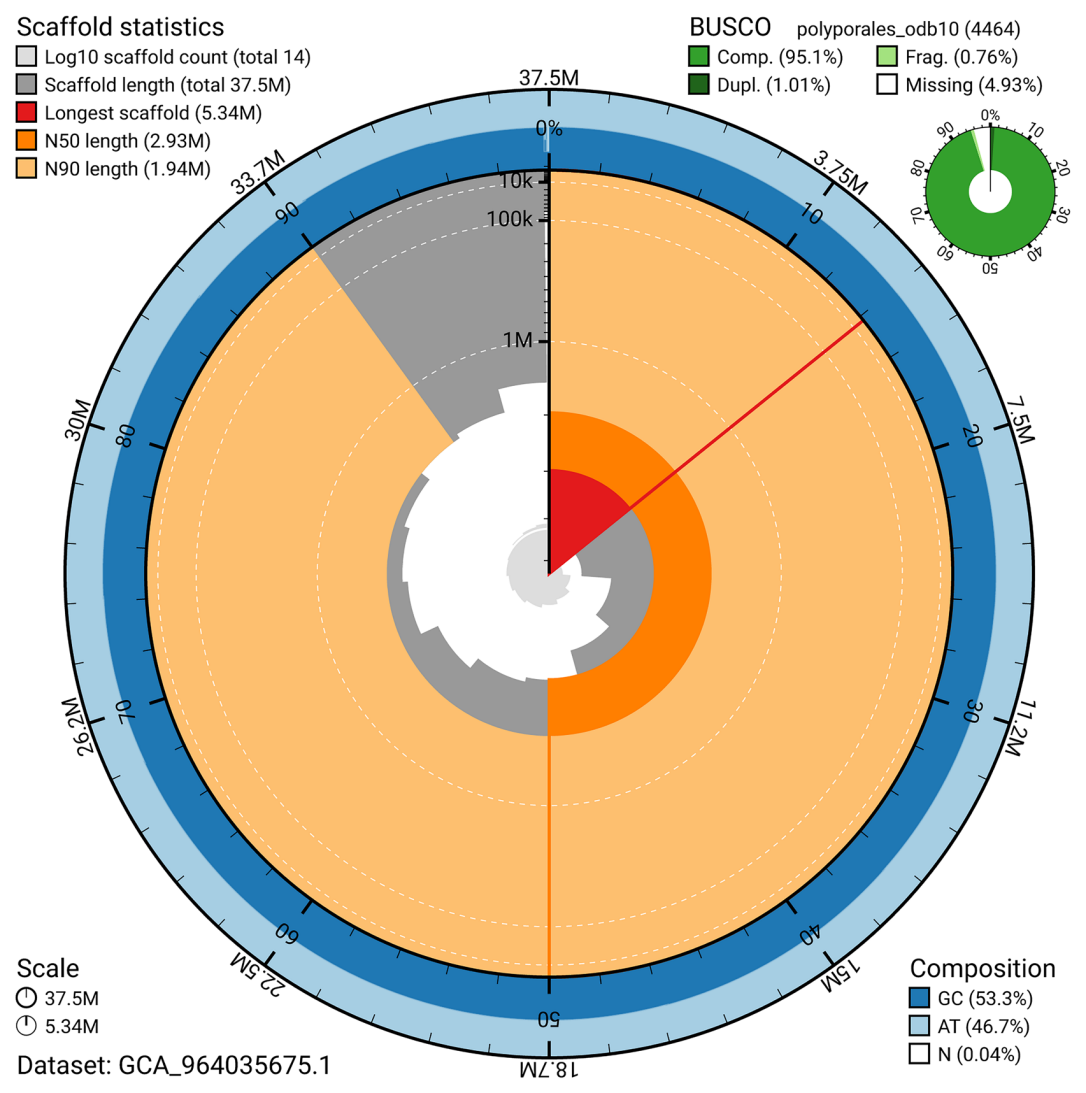
Genome assembly of *Buglossoporus quercinus*, gfBolQuer1.1: metrics. The BlobToolKit snail plot provides an overview of assembly metrics and BUSCO gene completeness. The circumference represents the length of the whole genome sequence, and the main plot is divided into 1,000 bins around the circumference. The outermost blue tracks display the distribution of GC, AT, and N percentages across the bins. Scaffolds are arranged clockwise from longest to shortest and are depicted in dark grey. The longest scaffold is indicated by the red arc, and the deeper orange and pale orange arcs represent the N50 and N90 lengths. A light grey spiral at the centre shows the cumulative scaffold count on a logarithmic scale. A summary of complete, fragmented, duplicated, and missing BUSCO genes in the polyporales_odb10 set is presented at the top right. An interactive version of this figure is available at https://blobtoolkit.genomehubs.org/view/GCA_964035675.1/dataset/GCA_964035675.1/snail.

**Figure 4.**
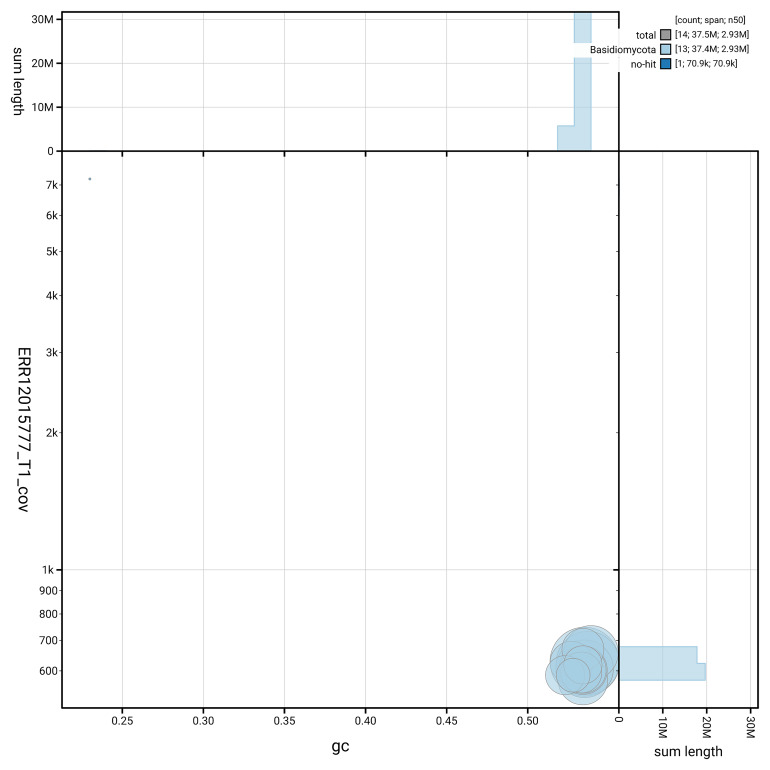
Genome assembly of *Buglossoporus quercinus*, gfBolQuer1.1: BlobToolKit GC-coverage plot. Blob plot showing sequence coverage (vertical axis) and GC content (horizontal axis). The circles represent scaffolds, with the size proportional to scaffold length and the colour representing phylum membership. The histograms along the axes display the total length of sequences distributed across different levels of coverage and GC content. An interactive version of this figure is available at https://blobtoolkit.genomehubs.org/view/GCA_964035675.1/blob.

The snail plot in Figure 3 provides a summary of the assembly statistics, indicating the distribution of scaffold lengths and other assembly metrics. Figure 4 shows the distribution of scaffolds by GC proportion and coverage.

**Figure 5.**
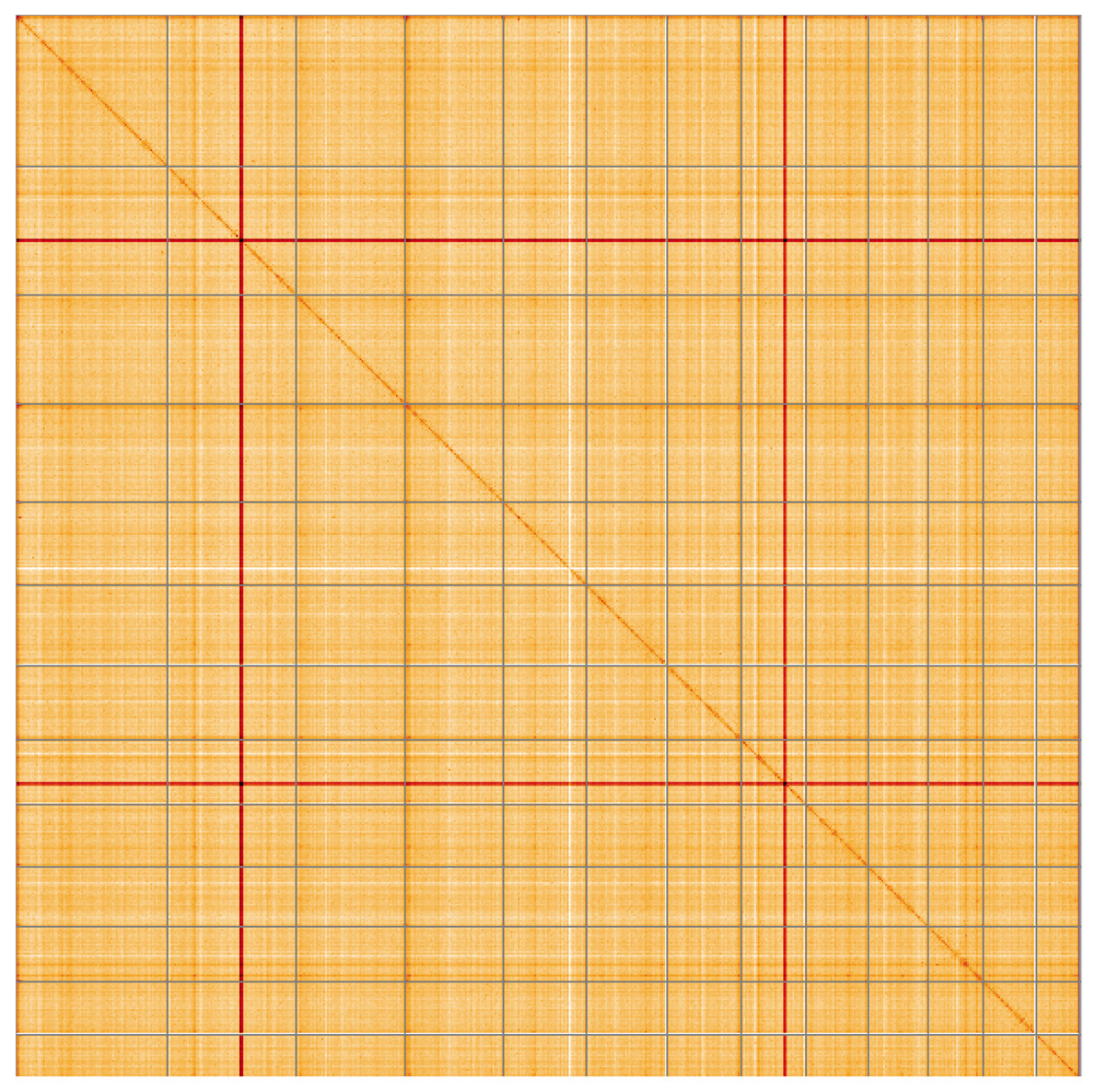
Genome assembly of *Buglossoporus quercinus*, gfBolQuer1.1: Hi-C contact map of the gfBolQuer1.1 assembly, visualised using HiGlass. Chromosomes are shown in order of size from left to right and top to bottom. An interactive version of this figure may be viewed at https://genome-note-higlass.tol.sanger.ac.uk/l/?d=cmkCv6z6RbCR5TA2g4GS0Q.

Most of the assembly sequence (99.81%) was assigned to 13 chromosomal-level scaffolds. These chromosome-level scaffolds, confirmed by Hi-C data, are named according to size ( Figure 5; Table 3).

**Table 3.** Chromosomal pseudomolecules in the genome assembly of *Buglossoporus quercinus*, gfBolQuer1.

INSDC accession	Name	Length (Mb)	GC%
OZ037956.1	1	5.34	53.5
OZ037957.1	2	4.52	53.5
OZ037958.1	3	3.84	53.5
OZ037959.1	4	3.46	54
OZ037960.1	5	2.93	53.5
OZ037961.1	6	2.84	53.5
OZ037962.1	7	2.61	53.5
OZ037963.1	8	2.28	52.5
OZ037964.1	9	2.19	53.5
OZ037965.1	10	2.1	53.5
OZ037966.1	11	1.94	52.5
OZ037967.1	12	1.87	53.5
OZ037968.1	13	1.52	53
OZ037969.1	MT	0.07	23

The mitochondrial genome was also assembled. This sequence is included as a contig in the multifasta file of the genome submission and as a standalone record in GenBank.

### Assembly quality metrics

The estimated Quality Value (QV) and *k*-mer completeness metrics, along with BUSCO completeness scores, were calculated for each haplotype and the combined assembly. The QV reflects the base-level accuracy of the assembly, while *k*-mer completeness indicates the proportion of expected *k*-mers identified in the assembly. BUSCO scores provide a measure of completeness based on benchmarking universal single-copy orthologues.

The primary haplotype has a QV of 64.7, the alternate haplotype 66.9, and the combined primary and alternate assemblies achieve an estimated QV of 65.6. The *k*-mer completeness for the primary haplotype is 88.76%, and for the alternate haplotype it is 86.47%. The combined primary and alternate assemblies achieve a *k*-mer completeness of 98.85%. BUSCO analysis using the polyporales_odb10 reference set ( *n* = 4,464) indicated a completeness score of 95.1% (single = 94.1%, duplicated = 1.0%).

Table 2 provides assembly metric benchmarks adapted from Rhie *et al.* (2021) and the Earth BioGenome Project (EBP) Report on Assembly Standards September 2024. The assembly achieves the EBP reference standard of **6.C.Q64.**

## Methods

### Sample acquisition

A specimen of *Buglossoporus quercinus* (specimen ID KDTOL00149, ToLID gfBolQuer1) was collected from Cardiff University, Cardiff, Wales, United Kingdom (latitude 51.49, longitude –3.18) on 2021-07-19. This was grown in culture and then collected into tubes and snap-frozen for shipping. The specimen was collected by Martha Crockatt (Cardiff University) and Richard Wright (Forever Fungi) and identified by Martha Crockatt, Richard Wright, Brian Douglas (Royal Botanic Gardens Kew) and Kieran Woof (Royal Botanic Gardens Kew). Metadata collection for samples adhered to the Darwin Tree of Life project standards described by Lawniczak *et al.* (2022).

### Nucleic acid extraction

The workflow for high molecular weight (HMW) DNA extraction at the WSI Tree of Life Core Laboratory includes sample preparation and homogenisation, DNA extraction, fragmentation and purification. Detailed protocols are available on protocols.io ( Denton *et al.*, 2023b). The gfBolQuer1 sample was weighed and dissected on dry ice ( Jay *et al.*, 2023) and tissue from mycelium was homogenised using a PowerMasher II tissue disruptor ( Denton *et al.*, 2023a). HMW DNA was extracted using the Automated Plant MagAttract v1 protocol ( Sheerin *et al.*, 2023). HMW DNA was sheared into an average fragment size of 12–20 kb in a Megaruptor 3 system ( Todorovic *et al.*, 2023). Sheared DNA was purified by solid-phase reversible immobilisation, using AMPure PB beads to eliminate shorter fragments and concentrate the DNA ( Strickland *et al.*, 2023). The concentration of the sheared and purified DNA was assessed using a Nanodrop spectrophotometer and Qubit Fluorometer and Qubit dsDNA High Sensitivity Assay kit. Fragment size distribution was evaluated by running the sample on the FemtoPulse system.

RNA was extracted from mycelium tissue of gfBolQuer1 in the Tree of Life Laboratory at the WSI using the RNA Extraction: Automated MagMax™ *mir*Vana protocol ( do Amaral *et al.*, 2023). The RNA concentration was assessed using a Nanodrop spectrophotometer and a Qubit Fluorometer using the Qubit RNA Broad-Range Assay kit. Analysis of the integrity of the RNA was done using the Agilent RNA 6000 Pico Kit and Eukaryotic Total RNA assay.

### Library preparation and sequencing

Library preparation and sequencing were performed at the WSI Scientific Operations core.

***PacBio HiFi***

The sample requires Covaris g-TUBE shearing to approximately 10 kb prior to library preparation. Ultra-low input libraries were prepared using PacBio SMRTbell® Express Template Prep Kit 2.0 and PacBio SMRTbell® gDNA Sample Amplification Kit. To begin, samples were normalised to 20 ng of DNA. Initial removal of single-strand overhangs, DNA damage repair, and end repair/A-tailing were performed per manufacturer’s instructions. From the SMRTbell® gDNA Sample Amplification Kit, amplification adapters were then ligated. A 0.85X pre-PCR clean-up was performed with Promega ProNex beads and the sample was then divided into two for a dual PCR. PCR reactions A and B each followed the PCR programs as described in the manufacturer’s protocol. A 0.85X post-PCR clean-up was performed with ProNex beads for PCR reactions A and B and DNA concentration was quantified using the Qubit Fluorometer v4.0 (Thermo Fisher Scientific) and Qubit HS Assay Kit and fragment size analysis was carried out using the Agilent Femto Pulse Automated Pulsed Field CE Instrument (Agilent Technologies) and gDNA 55kb BAC analysis kit. PCR reactions A and B were then pooled, ensuring the total mass was ≥500 ng in 47.4 μl. The pooled sample then repeated the process for DNA damage repair, end repair/A-tailing and additional hairpin adapter ligation. A 1X clean-up was performed with ProNex beads and DNA concentration was quantified using the Qubit and fragment size analysis was carried out using the Agilent Femto Pulse Automated Pulsed Field CE Instrument (Agilent Technologies). Size selection was performed using Sage Sciences' PippinHT system with target fragment size determined by analysis from the Femto Pulse, usually a value between 4000 and 9000 bp. Size selected libraries were then cleaned-up using 1.0X ProNex beads and normalised to 2 nM before proceeding to sequencing.

Samples were sequenced on a Sequel IIe instrument (Pacific Biosciences, California, USA). The concentration of the library loaded onto the Sequel IIe was in the range 40–135 pM. The SMRT link software, a PacBio web-based end-to-end workflow manager, was used to set-up and monitor the run, as well as perform primary and secondary analysis of the data upon completion.

***Hi-C***

Hi-C data were generated from mycelium tissue of gfBolQuer1 using the Arima-HiC v2 kit at the WSI Scientific Operations core. Tissue was finely ground using cryoPREP and then subjected to nuclei isolation. Nuclei were isolated using a modified protocol of the Qiagen QProteome Cell Compartment Kit where only CE1 and CE2 buffers are used in combination with QiaShredder spin columns. After isolation, the nuclei were fixed using 37% formaldehyde solution to crosslink the DNA. The crosslinked DNA was then digested using the restriction enzyme master mix. The 5’-overhangs were then filled in and labelled with biotinylated nucleotides and proximally ligated. An overnight incubation was carried out for enzymes to digest remaining proteins and for crosslinks to reverse. A clean up was performed with SPRIselect beads prior to library preparation. DNA concentration was quantified using the Qubit Fluorometer v4.0 and Qubit HS Assay Kit according to the manufacturer’s instructions.

For Hi-C library preparation, DNA was fragmented to a size of 400 to 600 bp using a Covaris E220 sonicator. The DNA was then enriched, barcoded, and amplified using the NEBNext Ultra II DNA Library Prep Kit (New England Biolabs) following manufacturer’s instructions. Hi-C sequencing was performed using paired-end sequencing with a read length of 150 bp on an Illumina NovaSeq 6000 instrument.

***RNA***

Poly(A) RNA-Seq libraries were constructed using the NEB Ultra II RNA Library Prep kit, following the manufacturer’s instructions. RNA sequencing was performed on the Illumina NovaSeq 6000 instrument.

### Genome assembly, curation and evaluation

***Assembly***

Prior to assembly of the PacBio HiFi reads, a database of *k*-mer counts ( *k* = 31) was generated from the filtered reads using FastK. GenomeScope2 ( Ranallo-Benavidez *et al.*, 2020) was used to analyse the *k*-mer frequency distributions, providing estimates of genome size, heterozygosity, and repeat content.

The HiFi reads were assembled using Hifiasm in the original mode ( Cheng *et al.*, 2021). Haplotypic duplications were identified and removed using purge_dups ( Guan *et al.*, 2020). The Hi-C reads were mapped to the primary contigs using bwa-mem2 ( Vasimuddin *et al.*, 2019). The contigs were further scaffolded using the provided Hi-C data ( Rao *et al.*, 2014) in YaHS ( Zhou *et al.*, 2023) using the --break option for handling potential misassemblies. The scaffolded assemblies were evaluated using Gfastats ( Formenti *et al.*, 2022), BUSCO ( Manni *et al.*, 2021) and MERQURY.FK ( Rhie *et al.*, 2020).

The mitochondrial genome was assembled using MitoHiFi ( Uliano-Silva *et al.*, 2023).

***Assembly curation***

The assembly was decontaminated using the Assembly Screen for Cobionts and Contaminants (ASCC) pipeline (article in preparation). Flat files and maps used in curation were generated in TreeVal ( Pointon *et al.*, 2023). Manual curation was primarily conducted using PretextView ( Harry, 2022), with additional insights provided by JBrowse2 ( Diesh *et al.*, 2023) and HiGlass ( Kerpedjiev *et al.*, 2018). Scaffolds were visually inspected and corrected as described by Howe *et al.* (2021). Any identified contamination, missed joins, and mis-joins were corrected, and duplicate sequences were tagged and removed. The process is documented at https://gitlab.com/wtsi-grit/rapid-curation (article in preparation).

***Assembly quality evaluation***

The Merqury.FK tool ( Rhie *et al.*, 2020) was used to evaluate *k*-mer completeness and assembly quality for the primary and alternate haplotypes using the *k*-mer databases ( *k* = 31) computed prior to genome assembly. The analysis outputs included assembly QV scores and completeness statistics.

A Hi-C contact map was produced for the final version of the assembly. The Hi-C reads were aligned using bwa-mem2 ( Vasimuddin *et al.*, 2019) and the alignment files were combined using SAMtools ( Danecek *et al.*, 2021). The Hi-C alignments were converted into a contact map using BEDTools ( Quinlan & Hall, 2010) and the Cooler tool suite ( Abdennur & Mirny, 2020). The contact map was visualised in HiGlass ( Kerpedjiev *et al.*, 2018).

The blobtoolkit pipeline is a Nextflow port of the previous Snakemake Blobtoolkit pipeline ( Challis *et al.*, 2020). It aligns the PacBio reads in SAMtools and minimap2 ( Li, 2018) and generates coverage tracks for regions of fixed size. In parallel, it queries the GoaT database ( Challis *et al.*, 2023) to identify all matching BUSCO lineages to run BUSCO ( Manni *et al.*, 2021). For the three domain-level BUSCO lineages, the pipeline aligns the BUSCO genes to the UniProt Reference Proteomes database ( Bateman *et al.*, 2023) with DIAMOND ( Buchfink *et al.*, 2021) blastp. The genome is also split into chunks according to the density of the BUSCO genes from the closest taxonomic lineage, and each chunk is aligned to the UniProt Reference Proteomes database with DIAMOND blastx. Genome sequences with no hits are chunked with seqtk and aligned to the NT database with blastn ( Altschul *et al.*, 1990). The blobtools suite combines all these outputs into a blobdir for visualisation.

The blobtoolkit pipeline was developed using nf-core tooling ( Ewels *et al.*, 2020) and MultiQC ( Ewels *et al.*, 2016), relying on the Conda package manager, the Bioconda initiative ( Grüning *et al.*, 2018), the Biocontainers infrastructure ( da Veiga Leprevost *et al.*, 2017), as well as the Docker ( Merkel, 2014) and Singularity ( Kurtzer *et al.*, 2017) containerisation solutions.

Table 4 contains a list of relevant software tool versions and sources.

**Table 4.** Software tools: versions and sources.

Software tool	Version	Source
BEDTools	2.30.0	https://github.com/arq5x/bedtools2
BLAST	2.14.0	ftp://ftp.ncbi.nlm.nih.gov/blast/executables/blast+/
BlobToolKit	4.3.9	https://github.com/blobtoolkit/blobtoolkit
BUSCO	5.5.0	https://gitlab.com/ezlab/busco
bwa-mem2	2.2.1	https://github.com/bwa-mem2/bwa-mem2
Cooler	0.8.11	https://github.com/open2c/cooler
DIAMOND	2.1.8	https://github.com/bbuchfink/diamond
fasta_windows	0.2.4	https://github.com/tolkit/fasta_windows
FastK	427104ea91c78c3b8b8b49f1a7d6bbeaa869ba1c	https://github.com/thegenemyers/FASTK
Gfastats	1.3.6	https://github.com/vgl-hub/gfastats
GoaT CLI	0.2.5	https://github.com/genomehubs/goat-cli
Hifiasm	0.16.1-r375	https://github.com/chhylp123/hifiasm
HiGlass	44086069ee7d4d3f6f3f0012569789ec138f42b84aa4 4357826c0b6753eb28de	https://github.com/higlass/higlass
MerquryFK	d00d98157618f4e8d1a9190026b19b471055b22e	https://github.com/thegenemyers/MERQURY.FK
Minimap2	2.24-r1122	https://github.com/lh3/minimap2
MitoHiFi	2	https://github.com/marcelauliano/MitoHiFi
MultiQC	1.14, 1.17, and 1.18	https://github.com/MultiQC/MultiQC
NCBI Datasets	15.12.0	https://github.com/ncbi/datasets
Nextflow	23.10.0	https://github.com/nextflow-io/nextflow
PretextView	0.2.5	https://github.com/sanger-tol/PretextView
purge_dups	1.2.3	https://github.com/dfguan/purge_dups
samtools	1.19.2	https://github.com/samtools/samtools
sanger-tol/ascc	-	https://github.com/sanger-tol/ascc
sanger-tol/blobtoolkit	0.5.1	https://github.com/sanger-tol/blobtoolkit
Seqtk	1.3	https://github.com/lh3/seqtk
Singularity	3.9.0	https://github.com/sylabs/singularity
TreeVal	1.2.0	https://github.com/sanger-tol/treeval
YaHS	1.1a.2	https://github.com/c-zhou/yahs

### Wellcome Sanger Institute – Legal and Governance

The materials that have contributed to this genome note have been supplied by a Darwin Tree of Life Partner. The submission of materials by a Darwin Tree of Life Partner is subject to the **‘Darwin Tree of Life Project Sampling Code of Practice’**, which can be found in full on the Darwin Tree of Life website here. By agreeing with and signing up to the Sampling Code of Practice, the Darwin Tree of Life Partner agrees they will meet the legal and ethical requirements and standards set out within this document in respect of all samples acquired for, and supplied to, the Darwin Tree of Life Project.

Further, the Wellcome Sanger Institute employs a process whereby due diligence is carried out proportionate to the nature of the materials themselves, and the circumstances under which they have been/are to be collected and provided for use. The purpose of this is to address and mitigate any potential legal and/or ethical implications of receipt and use of the materials as part of the research project, and to ensure that in doing so we align with best practice wherever possible. The overarching areas of consideration are:

•    Ethical review of provenance and sourcing of the material

•    Legality of collection, transfer and use (national and international)

Each transfer of samples is further undertaken according to a Research Collaboration Agreement or Material Transfer Agreement entered into by the Darwin Tree of Life Partner, Genome Research Limited (operating as the Wellcome Sanger Institute), and in some circumstances other Darwin Tree of Life collaborators.

## Data Availability

European Nucleotide Archive: Buglossoporus quercinus (oak polypore). Accession number PRJEB65737; https://identifiers.org/ena.embl/PRJEB65737. The genome sequence is released openly for reuse. The *Buglossoporus quercinus* genome sequencing initiative is part of the Darwin Tree of Life (DToL) project. All raw sequence data and the assembly have been deposited in INSDC databases. The genome will be annotated using available RNA-Seq data and presented through the Ensembl pipeline at the European Bioinformatics Institute. Raw data and assembly accession identifiers are reported in Table 1.

## Author information

Members of the Royal Botanic Gardens Kew Genome Acquisition Lab are listed here: https://doi.org/10.5281/zenodo.12625079.

Members of the Wellcome Sanger Institute Tree of Life Management, Samples and Laboratory team are listed here: https://doi.org/10.5281/zenodo.12162482.

Members of Wellcome Sanger Institute Scientific Operations: Sequencing Operations are listed here: https://doi.org/10.5281/zenodo.12165051.

Members of the Wellcome Sanger Institute Tree of Life Core Informatics team are listed here: https://doi.org/10.5281/zenodo.12160324.

Members of the Tree of Life Core Informatics collective are listed here: https://doi.org/10.5281/zenodo.12205391.

Members of the Darwin Tree of Life Consortium are listed here: https://doi.org/10.5281/zenodo.4783558.
