# Supplementary material for: The genome sequence of the Oak Polypore, Buglossoporus quercinus (Schrad.) Kotl. & Pouzar
Source: Wellcome Open Res. 2025 Aug 22;10:456. [Version 1] doi: 10.12688/wellcomeopenres.24714.1 (PMC12498085; doi:10.12688/wellcomeopenres.24714.1)
Supplement: Peer review history

# Reviewer report: Reviewer response for version 1

**DOI:** 10.21956/wellcomeopenres.27230.r132668

**Published:** 2025-10-06

**Authors:** Saleh Rahimlou

**Affiliations:** 1 University of Michigan, Ann Arbor, Michigan, USA

This is a well-prepared manuscript that presents a high-quality genome for an important Polypore species. The work is carefully executed and the data thoroughly analyzed. That said, I find two important aspects missing. First, although RNA-seq data was generated, it is not clear whether it was utilized for genome annotation. Including annotation statistics (e.g., predicted numbers of genes, exons, introns, and coding sequences) would substantially strengthen the manuscript. Second, a comparative perspective is lacking. How does this genome compare to other members of the Fomitopsidaceae, such as Fomitopsis, in terms of genome size, GC content, and predicted protein-coding gene content? Providing this context would enhance the impact of the study.

 Figure 4 (blobplot) has quite a lot of white space, likely caused by a single short contig/scaffold with very low GC content and unusually high coverage. I’m curious what this contig represents—could it be contamination, or perhaps a bacterial endosymbiont? I recommend examining this contig first. BlobToolKit also allows refining taxonomy to lower levels if it is not a spread across irrelevant taxa. Additionally, please correct the y-axis label.

 The text doesn’t have the line numbers to refer, and it looks like it is prepared for indexing. Anyway, the underlined part of this paragraph is redundant.

 The blobtoolkit pipeline was developed using nf-core tooling (Ewels et al., 2020) and MultiQC (Ewels et al., 2016), r elying on the Conda package manager, the Bioconda initiative (Grüning et al., 2018), the Biocontainers infrastructure (da Veiga Leprevost et al., 2017), as well as the Docker (Merkel, 2014) and Singularity (Kurtzer et al., 2017) containerization solutions.

 The blobtoolkit pipeline is a Nextflow port of the previous Snakemake Blobtoolkit pipeline (Challis et al., 2020). It aligns the PacBio reads in SAMtools and minimap2 (Li, 2018) and generates coverage tracks for regions of fixed size.

 The sentence does not make sense. Probably you mean the PacBio raw reads aligned to the assembly using minimap2. SAMtools is not an alignment tool. Probably it is used to index and sort the alignment file (sam/bam file).

Are sufficient details of methods and materials provided to allow replication by others?

Yes

Is the rationale for creating the dataset(s) clearly described?

Yes

Are the datasets clearly presented in a useable and accessible format?

Yes

Are the protocols appropriate and is the work technically sound?

Yes

Reviewer Expertise:

Fungal genomics, metagenomics and comparative phylogenetic analyses

I confirm that I have read this submission and believe that I have an appropriate level of expertise to confirm that it is of an acceptable scientific standard.

# Reviewer report: Reviewer response for version 1

**DOI:** 10.21956/wellcomeopenres.27230.r132675

**Published:** 2025-09-29

**Authors:** Essy Harnelly

**Affiliations:** 1 Universitas Syiah Kuala, Banda Aceh, Indonesia

Overall, the manuscript is well written, transparent, and the data are of high quality.

 This article will enrich the reference for researchers interested in fungal conservation and genomics.

 This article presents a chromosome-level assembly of the oak polypore fungus *Buglossoporus quercinus*, generated as part of the Darwin Tree of Life project. The authors provide a clear background on the species’ conservation significance and report an impressive set of sequencing data (PacBio HiFi, Hi-C, RNA-Seq) yielding a 37.43 Mb genome scaffolded into 13 chromosomal pseudomolecules covering 99.81 % of the assembly, with a mitochondrial genome assembled separately. The reported assembly metrics (QV > 64, BUSCO completeness 95.1 %) meet or exceed the Earth BioGenome Project reference standard 6.C.Q64. All raw data and assemblies are deposited in INSDC databases, software versions and protocols are openly cited, and interactive views of the assembly are provided. These aspects make the dataset highly reproducible and a valuable resource for fungal conservation genetics.

 I have only minor suggestions for this article:

1. Please provide at least a minimal overview of the predicted gene models, or state clearly that annotation is forthcoming via Ensembl 
2. Could you consider adding a short paragraph comparing genome size, GC content, or BUSCO scores with related polypores (e.g. *Fomitopsis betulina*, *Laetiporus sulphureus*) would help readers place this genome in context.
3. Please expand figure captions (especially Figures 1 and 2) with clearer descriptions of each panel and scale bar and correct minor typographical errors (e.g. “sprocarp” to “sporocarp”).
4. Given the mention of heterozygosity and inbreeding risk, it would be beneficial to include a sentence explaining how this reference genome could support future population genomics or translocation planning.

Recommendation: Accept with minor revision

Are sufficient details of methods and materials provided to allow replication by others?

Yes

Is the rationale for creating the dataset(s) clearly described?

Yes

Are the datasets clearly presented in a useable and accessible format?

Partly

Are the protocols appropriate and is the work technically sound?

Yes

Reviewer Expertise:

Plant genetics

I confirm that I have read this submission and believe that I have an appropriate level of expertise to confirm that it is of an acceptable scientific standard.
